# Steady-State Somatosensory Evoked Potential for Brain-Computer Interface—Present and Future

**DOI:** 10.3389/fnhum.2015.00716

**Published:** 2016-01-14

**Authors:** Sangtae Ahn, Kiwoong Kim, Sung Chan Jun

**Affiliations:** ^1^School of Information and Communications, Gwangju Institute of Science and TechnologyGwangju, South Korea; ^2^Center for Biosignals, Korea Research Institute of Standards and ScienceDaejeon, South Korea; ^3^Department of Medical Physics, University of Science and TechnologyDaejeon, South Korea

**Keywords:** steady-state somatosensory evoked potential, brain-computer interface, vibrotactile stimulation, tactile selective attention, perceptual load

## Abstract

Brain-computer interface (BCI) performance has achieved continued improvement over recent decades, and sensorimotor rhythm-based BCIs that use motor function have been popular subjects of investigation. However, it remains problematic to introduce them to the public market because of their low reliability. As an alternative resolution to this issue, visual-based BCIs that use P300 or steady-state visually evoked potentials (SSVEPs) seem promising; however, the inherent visual fatigue that occurs with these BCIs may be unavoidable. For these reasons, steady-state somatosensory evoked potential (SSSEP) BCIs, which are based on tactile selective attention, have gained increasing attention recently. These may reduce the fatigue induced by visual attention and overcome the low reliability of motor activity. In this literature survey, recent findings on SSSEP and its methodological uses in BCI are reviewed. Further, existing limitations of SSSEP BCI and potential future directions for the technique are discussed.

## Introduction

Brain-computer interface (BCI) has been investigated widely in neuroscience, and has attracted considerable attention as a promising emerging technology since, Vidal (1973) first introduced BCI as a method to interface between humans and computers or machines. In three decades, a BCI system that employs visual attention and exhibits high performance has been commercialized (www.intendix.com); the system has been demonstrated to decode 5–10 characters per min. Due to the continual efforts of many researchers, BCI systems with far higher information transfer rates than those that exist now will be on the market soon. Visual-based BCIs that use P300 and steady-state visually evoked potential (SSVEP) also have entered the mainstream since their numerous novel experimental paradigms and methodological approaches have been reported and published (Vialatte et al., 2010; Fazel-Rezai et al., 2012). Visual-based BCIs are known to have certain advantages, in that they are highly reliable and are easy to set up in experimental paradigms. Recently, open-source software for BCI research (Renard et al., 2010) has been developed that provides well-designed P300 and SSVEP scenarios for diverse applications. Visual-based BCIs, however, need to be modified to reduce training time (Rivet et al., 2011) and the cognitive workload that results in fatigue after prolonged use (Käthner et al., 2014); this may be one of the greatest challenges that face visual-based BCIs.

In addition to visual-based BCIs, sensorimotor rhythm (SMR)-based BCIs also have been investigated thoroughly. In the literature (Yuan and He, 2014), modulations in sensorimotor cortex by actual movement and motor intention or motor imagery may yield a control signal that enables a person to operate the machines and control the cursor in up to three dimensions. These SMR-based BCIs are quite compelling for paralyzed users (Birbaumer et al., 2008), and are used to expedite motor rehabilitation and recovery in order to enhance motor function (Pascual-Leone et al., 2005). For example, it has been reported that patients who suffered strokes recovered their motor ability through rehabilitation and were able to control grasping actions through a mechanical hand orthosis without any limb movements (Buch et al., 2008). However, there are still many obstacles to overcome before SMR-based BCIs will be reliable and available for universal use. One of the most challenging issues includes inherent inter- and intra-subject variations in performance, as reported in the recent literature (Ahn et al., 2013a,b; Cho et al., 2015); thus, a significant number of users have great difficulty controlling the system, a condition referred to as “BCI illiteracy”. This may be correlated with psychological, anatomical, and physiological factors (Ahn and Jun, 2015).

A potential approach to resolve the issues that confront BCI development is to employ tactile sensation. By comparison to visual stimulation, tactile sensation produces less visual fatigue and can be utilized for patients who cannot gaze at the flickering lights consistently. Generally, the mechanoreceptors in human glabrous skin consist of four distinct elements—Merkel, Meissner, Pacinian, and Ruffini cells. Among them, Meissner corpuscles lie in the tips of the dermal papillae adjacent to the primary ridges and closest to the skin surface. These are particularly efficient in transducing information with low-frequency vibrations (1–40 Hz) and thus, play a central role in detecting sensory vibration (Purves et al., 2012). Based on the processing of tactile sensation, one study (Müller-Putz et al., 2006) attempted to demonstrate the suitability of tactile-based BCI using stimulation of the index fingers. A resonance peak in the given frequency interval was extracted with a locked-in analyzer system that allowed the subjects’ tactile selective attention to be decoded. In other words, subjects are instructed to intend or attend one target stimulation between two simultaneous stimulations; thus it is observed that spectral amplitudes of target trials are notably greater than those of non-target trials. This phenomenon may be introduced to classify subjects’ intention. Even though the classification accuracy was reported to be approximately 70%, the feasibility of BCI with steady-state somatosensory evoked potential (SSSEP) and tactile selective attention was first exploited. This SSSEP can be applied in particular to patients with locked-in syndrome or amyotrophic lateral sclerosis, as they are still able to modulate brain activity using their somatosensory system (Cosi et al., 1984; Soria et al., 1989), although it may not be useful to complete spinal cord injury patients due to their loss of sensory related functions. Other studies have investigated this technique since then, but no notable results have been reported. This may be because it is quite challenging to set up tactile stimulation hardware that will generate reasonable sinusoidal or modulated stimulation patterns (Pokorny et al., 2014). Even though there is great potential for SSSEP to yield improved BCI systems, to the best of our knowledge, there have been few in-depth studies of SSSEP and its application in BCI. Therefore, in this article, the majority of the studies of SSSEP and various BCI approaches that use it are reviewed in detail. In addition, the limitations of SSSEP BCI and possible future directions for its use are discussed.

## SSSEP Studies

In this section, we summarize several SSSEP related works, which are listed in Table 1. Characterization of SSSEP was first introduced by Snyder (1992). He applied amplitude-modulated (AM) vibrations to the fingers and palms, and attempted to find a stimulation frequency within 2–43 Hz that yielded the highest signal-to-noise ratio (SNR) evoked potential. He found that stimulation at 26 Hz produced the highest SNR among them. This was the first step in producing non-transient SSSEP responses in the effort to determine an appropriate frequency range of stimulation. Thereafter, Noss et al. (1996) reported the advantages of a steady-state compared to a transient response. They applied AM electrical alternating current waveforms to the left median nerve and compared its speed and reliability to that of the transient response; modulation frequencies of 7.4, 14.7, 25.6, and 41.2 Hz were used for AM stimulations. Significant contra-lateral peaks (*p* < 0.01) at a stimulation frequency of 25.6 Hz were observed using only a 10 s epoch, which was faster than transient pulse stimulation in a time-domain analysis, and thus, demonstrated the possibility of using the steady-state somatosensory response. Because vibratory stimulation was suitable in the analysis of the steady-state response, Tobimatsu et al. (1999) custom-built a stimulator and applied mechanical vibrations from 2–30 Hz to the right palmar surface. A carrier frequency of 128 Hz and modulation frequencies of 5, 7, 11, 14, 15, 17, 21, 25, and 30 Hz with a fixed intensity of 0.05 newton (N) were introduced. Among the nine modulation frequencies, 21 Hz yielded the highest peak, and the first harmonic component was more predominant than was the second. In addition, when the stimulus intensity varied from 0.001–0.1N (0.001, 0.003, 0.008, 0.01, 0.03, 0.05, 0.08, and 0.1) at a fixed, 21 Hz modulation frequency, they found relatively smaller peaks at lower intensities that were visually discriminable from noise. The mean amplitudes of eight stimulation intensities reached a plateau at an intensity of 0.05 N (*p* < 0.0001). Therefore, they demonstrated clearly in this study that steady-state response amplitudes at 21 Hz and 0.05 N had the largest discriminable peaks. Followed by this study, vibratory stimuli were applied to the sole of the foot (Tobimatsu et al., 2000), and these authors demonstrated clearly that stimulation of the sole yielded an efficient and stable SSSEP comparable to stimulation of the palm. In addition to the amplitude behavior of SSSEP, Goto et al. (2003) calculated the coherence of frequency bands over channels and stimulations. The same vibratory stimulation reported in the literature (Tobimatsu et al., 1999, 2000) was applied to both palms, and coherence between lateralized channels (ipsi and contra-lateral) at the first harmonic frequency of stimulation was significantly lower than was that in the unstimulated condition. This demonstrated that vibratory stimulation resulted in inter-hemispheric desynchronization. A magnetoencephalography (MEG) and electroencephalography (EEG) study was performed by Nangini et al. (2006). A whole-head MEG device was used to elicit both transient and steady-state responses in the somatosensory cortex. Vibrotactile stimuli were applied to the right index finger at a stimulation frequency of 22 Hz, which is optimal for the somatosensory system and pneumatic stimulator. The authors found that the steady-state responses appeared in the contra-lateral primary somatosensory cortex, and based on localization, were spatially distinct from the transient responses. Thus, from the results above, we may infer that vibratory stimulations to glabrous skin, such as that on the palm and finger, can generate clear contra-lateral SSSEPs that are discriminated well compared to the unstimulated condition, regardless of modality.

**Table 1:** **Studies on SSSEP and BCI with SSSEP**.

Reference	Frequencies of stimulations (Hz)	Targets of stimulations	Major findings
**Studies on SSSEP**			
Snyder (1992)	2–40	Fingers and palm	Frequencies ~26 Hz produce the strongest signal. Inverse dipole modeling localized the somatosensory cortex.
Noss et al. (1996)	7.4, 14.7, 25.6, 41.2	Left median nerve	A reliable steady-state response can be recorded from scalp electrodes overlying the somatosensory cortex.
Tobimatsu et al. (1999)	5, 7, 11, 14, 15, 17, 21, 25, 30	Right palm	The highest peak occurs at 21 Hz in contra-lateral area. The amplitudes of the first harmonic exceed those of the second harmonic.
Tobimatsu et al. (2000)	17, 19, 21, 23, 25, 30	Right palm and sole	The amplitudes of the SSSEPs were highest in the contralateral hand and foot areas.
Goto et al. (2003)	21	Both palms	Coherence of the somatosensory area at 21 Hz was significantly lower than that in the unstimulated condition or intra-hemispheric coherence.
Nangini et al. (2006)	22	Right index finger	Dipoles associated with the steady-state responses were localized in two distinct regions within the primary somatosensory cortex.
Giabbiconi et al. (2004)	20, 26	Both index fingers	The amplitude of the frequency-coded SSSEP elicited by the vibration attended to was significantly greater when attention was focused on the respective finger.
(Giabbiconi et al., 2007)	20, 25	Both index fingers	Sustained spatial attention was mediated in the primary somatosensory cortex with no differences in SSSEP amplitude topographies between attended and unattended body locations.
**Studies on BCI with SSSEP**			
Müller-Putz et al. (2006)	17–35 (in 2 Hz steps)	Both index fingers	BCI system based on SSSEP was feasible.
Haegens et al. (2011)	25, 33, 41.7, 50, 66.7	Both thumbs	Pre-stimulus alpha lateralization in the somatosensory system behaved similarly to posterior alpha activity observed in visual attention tasks.
Breitwieser et al. (2012)	17–35 (in 2 Hz steps)	Right fingers	Person-specific resonance-like frequencies within 19–29 Hz were found. SSSEPs were classified with a hit rate from 51–96%.
Yao et al. (2013)	27	Both wrists	There was significant improvement from approximately 65% in motor imagery to over 80% in selective sensation in some subjects.
Yao et al. (2014)	27	Both wrists	Six subjects among eleven showed statistically significant improvement in hybrid modality, compared with either motor imagery or selective sensation alone.
Ahn et al. (2014)	16–25 (in 1 Hz steps)	Both thumbs	A proposed hybrid approach outperformed the others, yielding an approximately 10% improvement in classification accuracy compared to motor imagery alone.

To date, researchers have focused on extracting the properties of SSSEP and methodological approaches to achieving a reliable signal. Giabbiconi et al. (2004) investigated the properties of spatial attention using SSSEP; they found that when they stimulated both index fingers at different frequencies (20 and 26 Hz), and instructed subjects to attend to the stimulation of one finger associated with the direction indicated, and ignore stimulation of the other finger, SSSEP amplitude increased. Further, statistically significant SSSEP amplitudes in the contra-lateral frontal and fronto-central regions were observed. Subsequently, they attempted to pinpoint the precise region of the somatosensory cortex based on source localization with high-density EEG (Giabbiconi et al., 2007). Two stimulations (20 and 25 Hz) were applied to both index fingers, and they found bilateral activation of attention that was dominant in the primary somatosensory cortex. The secondary somatosensory cortex did not contribute to the SSSEP induced by attention. These two studies (Giabbiconi et al., 2004, 2007) demonstrated that a two-class task, such as left vs. right separation using tactile attention may be distinguishable. To summarize briefly, from studies of SSSEP, we can infer that the effective frequency range of stimulation is approximately 20–30 Hz, and the fingertip is the most sensitive skin on the body; thus, tactile spatial attention using a paradigm that incorporates simultaneous stimulation of two fingers with different frequencies yields discriminable and controllable information.

## BCI with SSSEP

**Figure 1:**
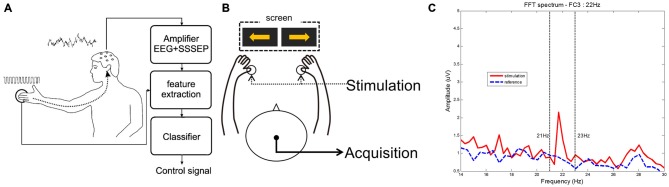
**Basic procedure and characteristics of steady-state somatosensory evoked potential (SSSEP) brain-computer interface (BCI) using tactile stimulation. (A)** Procedure of SSSEP BCI created by Müller-Putz et al. (2006); **(B)** Proposed experimental paradigm created by Ahn et al. (2014); **(C)** Power spectrum of right thumb stimulation between stimulation and reference created by Ahn et al. (2014).

In this section, we summarize several previous works on SSSEP with BCI, which are listed in Table 1. Müller-Putz et al. (2006) first investigated the feasibility of BCI with SSSEP. They defined the basic SSSEP-based BCI paradigm using resonance-like frequencies in order to control two classes (Müller et al., 2001). Figure 1A illustrates the entire procedure for SSSEP-based BCI in this study. They showed that the mean classification accuracy reached approximately 70% for five subjects, and that this novel BCI paradigm was applicable in an online environment, suggesting that SSSEP-based BCI, as well as visual-based, have great potential for future use. Thereafter, several studies of SSSEP BCI were presented as conference papers, but no significant findings were reported. Recently, an MEG study of a tactile discrimination task was attempted (Haegens et al., 2011). In this study, subjects were instructed to perform a somatosensory spatial attention task when both thumbs were stimulated simultaneously. Interestingly, they found a pre-stimulus alpha lateralization with respect to the direction of somatosensory attention, which may be applicable to the BCI system. In addition to the discrimination between the two hands, the fingers of one (right or left) hand were stimulated for the purposes of discrimination, as reported by Breitwieser et al. (2012). These authors conducted SSSEP BCI using all five fingers of the right hand with a carrier frequency of 200 Hz and a stimulation frequency that ranged between 17 and 35 Hz in 2 Hz steps. A Fisher’s linear discriminant analysis was applied for classification and again, the mean accuracy achieved was approximately 70%. In addition to stimulation of the fingers, Yao et al. (2013) applied stimulation to the skin on both wrists. They compared two paradigms, motor imagery, and selective sensation, and found that all participants achieved online classification accuracies of approximately 80%. They also combined two tasks in an attempt to improve classification performance (Yao et al., 2014). In this work, subjects performed a four class experiment; left/right selective sensation and left/right motor imagery. They used one condition in each task and the best combination was left selective sensation and right motor imagery. Lastly, our group achieved a hybrid BCI using motor imagery and tactile selective attention (Ahn et al., 2014). A custom-built stimulator was manufactured and validated in an SSSEP experiment as depicted in Figures 1B,C. Significant features of SSSEP were found and the feasibility of such features was tested to overcome the BCI illiteracy found in motor imagery. We demonstrated improved classification performance in motor imagery using tactile selective attention, and suggested effective methodological approaches for hybrid BCIs.

## Limitations and Future Directions

Studies related to SSSEP, as well as BCI approaches that use properties of SSSEP, were reviewed. Obviously, compared to visual or SMR-based BCI research (Vialatte et al., 2010; Yuan and He, 2014), only a few studies of SSSEP have been published. The relative paucity of such studies may be due to the following reasons: (1) It is difficult to use SSSEPs to discriminate attentional effects due to the difficulty of the tactile attention task; and (2) There is no well-designed standard tactile stimulator to elicit SSSEPs. A more detailed discussion of the limitations of SSSEP and their possible solutions follows.

### Perceptual Load in Tactile Selective Attention

A possible hurdle in SSSEP research is the difficulty of the tactile attention task. In our experience with SSSEP BCI experiments, it was difficult for subjects to attend to, and concentrate on, one of two simultaneous stimulations. In the questionnaire used in our previous work (Ahn et al., 2014), most subjects reported that they had some difficulty in attending to the stimulation of one finger when two were stimulated, by comparison to a conventional motor imagery or visual attention task. According to the perceptual load theory (Lavie, 2005), when a subject is fully attentive using full capacity in relevant attention processing, there may be no spare capacity for perception of distracting interference (high perceptual load). On the other hand, any capacity not allocated to the relevant attention processing may involuntarily spill over to the perception of distracting interference (low perceptual load). This hypothesis may be applicable not only to the visual modality (Sagi and Julesz, 1984; Schwartz et al., 2005), but to the somatosensory modality as well (Adler et al., 2009). Actually, when two synchronous stimulations are applied to the left and right fingers, as in conventional experimental paradigms, it is likely to disrupt the attention that can be focused on one fingertip stimulation alone, because vibrotactile stimulation with the same intensity and rhythmic pulse may not provide subjects with a clear discriminative cue. For this reason, Adler et al. (2009) tried to identify the difference between two different tactile attention tasks (detection and discrimination) to clarify the perceptual load hypothesis. The detection task (low perceptual load) was accomplished by applying both left and right tactile stimulus trains synchronously, similar to the conventional paradigm (Müller-Putz et al., 2006). The discrimination task (high perceptual load) included two types of stimuli (target and non-target) to elicit subjects’ full attention. They found that the SSSEP amplitude increased only in the discrimination task and the embedded transient response in that task was significantly higher than that in the detection task. With this reasoning, we should design the stimulation in a different way, such as applying a different Waltz rhythmic modulation for one of the two sources of stimulation. If two different rhythmic stimulations are applied to the fingers, subjects may be able to concentrate and attend to a specific stimulation more easily. For example, one Waltz rhythmic stimulation with 16 Hz and the other standard rhythmic stimulation with 21 Hz are applied to the left and right thumbs, respectively.

### Necessity for a Standardized Vibrotactile Stimulator

Another hurdle to overcome before BCI using SSSEP can be established is that there is no standard vibrotactile stimulator to elicit SSSEPs. Although it is quite laborious to construct a vibrotactile stimulator that is suitable for SSSEP, in most such investigations, researchers have custom-built the stimulators. Even though some commercial stimulators, such as g.STIMbox (g.tec medical engineering GmbH, Schiedlberg, Austria) and C-2 TACTOR (Engineering Acoustics Inc., Casselberry, FL, USA) are on the market, they are unable to generate a frequency less than one, as is necessary to extract the patterns of SSSEPs. To the best of our knowledge, no stimulator has been developed that can apply a stimulation frequency in fractional Hz steps, which is more comfortable for subjects and may be applied to various body parts. After tremendous time and effort in the preliminary stimulation and validation tests, we were finally successful in designing a stimulator that was able to elicit the SSSEP. As a possible solution for this problem, Pokorny et al. (2014) recently suggested a way to make a safe and reliable tactile stimulator. This is the first approach that has proposed several basic safety requirements and stimulation patterns for tactile-based experiments. The device proposed may be able to generate sinusoidal modulated waveforms based on different types of electromagnetic transducers, as well as various stimulation patterns that are stable in eliciting SSSEPs. This attempt suggests that we may be able to establish reliable, tactile-based BCIs and construct more easily stimulators that conform to a variety of requirements. Therefore, it is necessary for companies or leading research groups to develop, fabricate, and market a standardized vibrotactile stimulator.

## Conclusions

We surveyed previous works related to SSSEPs and BCIs using SSSEP. From this literature survey, we inferred that stimulation that ranges from 20–30 Hz at 0.05 N intensity is suitable to elicit a clear SSSEP, and the fingertips are the most sensitive part of the body. We also concluded that novel experimental paradigms must be developed to increase the reliability of SSSEP BCI, and SSSEP hybridization with other tasks, such as motor imagery. This is one of the most appealing approaches to improve the performance of these systems. To enhance the viability of tactile BCIs, we believe that well-designed, standardized vibrotactile stimulators and similar devices must be developed that will lead to effective experimental paradigms that allow subjects to concentrate more easily.

## Author Contributions

SA conducted the entire literature survey and wrote the manuscript. KK helped and consulted the literature survey. SCJ oversaw all procedures of the survey, discussed findings and insights from the survey, and wrote the manuscript.

## Conflict of Interest Statement

The authors declare that the research was conducted in the absence of any commercial or financial relationships that could be construed as a potential conflict of interest.
